# A Review of Flaviviruses that Have No Known Arthropod Vector

**DOI:** 10.3390/v9060154

**Published:** 2017-06-21

**Authors:** Bradley J. Blitvich, Andrew E. Firth

**Affiliations:** 1 Department of Veterinary Microbiology and Preventive Medicine, College of Veterinary Medicine, Iowa State University, Ames, IA 50011, USA; 2 Department of Pathology, University of Cambridge, Cambridge CB2 1QP, UK; aef24@cam.ac.uk

**Keywords:** flavivirus, no known vector, vertebrate-specific, bat, rodent, host range, transmission, genomic organization

## Abstract

Most viruses in the genus *Flavivirus* are horizontally transmitted between hematophagous arthropods and vertebrate hosts, but some are maintained in arthropod- or vertebrate-restricted transmission cycles. Flaviviruses maintained by vertebrate-only transmission are commonly referred to as no known vector (NKV) flaviviruses. Fourteen species and two subtypes of NKV flaviviruses are recognized by the International Committee on Taxonomy of Viruses (ICTV), and Tamana bat virus potentially belongs to this group. NKV flaviviruses have been isolated in nature almost exclusively from bats and rodents; exceptions are the two isolates of Dakar bat virus recovered from febrile humans and the recent isolations of Sokoluk virus from field-collected ticks, which raises questions as to whether it should remain classified as an NKV flavivirus. There is evidence to suggest that two other NKV flaviviruses, Entebbe bat virus and Yokose virus, may also infect arthropods in nature. The best characterized bat- and rodent-associated NKV flaviviruses are Rio Bravo and Modoc viruses, respectively, but both have received limited research attention compared to many of their arthropod-infecting counterparts. Herein, we provide a comprehensive review of NKV flaviviruses, placing a particular emphasis on their classification, host range, geographic distribution, replication kinetics, pathogenesis, transmissibility and molecular biology.

## 1. Introduction

All viruses in the genus *Flavivirus* (family *Flaviviridae*) possess a single-stranded, positive-sense RNA genome of 10–11 kb that encodes a 5′ untranslated region (5′ UTR), a long open reading frame (ORF) and a 3′ UTR [1]. The 5′ and 3′ UTRs normally consist of approximately 100 and 400–700 nucleotides (nt), respectively, and form highly conserved secondary and tertiary structures required for replication and translation [2]. The ORF encodes a large polyprotein that is further processed by viral and host proteases to generate three structural proteins, designated the capsid (C), premembrane/membrane (prM/M) and envelope (E) proteins and at least seven nonstructural (NS) proteins in the gene order: 5′-C–prM(M)–E–NS1–NS2A–NS2B–NS3–NS4A–2K–NS4B–NS5-3′ [1,3]. 

Despite a common genomic organization, flaviviruses possess fundamental differences in their natural host ranges and transmission cycles [4,5,6,7]. Most known flaviviruses are horizontally transmitted between hematophagous arthropods (i.e., mosquitoes and ticks) and vertebrate hosts; for example, dengue virus, yellow fever virus (YFV), Japanese encephalitis virus (JEV), West Nile virus (WNV), Zika virus (ZIKV) and tick-borne encephalitis virus (TBEV) [8,9,10]. Other flaviviruses have been isolated in nature exclusively from mosquitoes and sandflies and cannot replicate in vertebrate cell lines or suckling mice [11,12,13]. These viruses are assumed to have insect-restricted host ranges and are vertically transmitted between hosts [14,15]. Another group of viruses has been isolated in nature almost exclusively from vertebrates (bats, rodents and occasionally humans) and never from wild-caught or laboratory-inoculated arthropods or arthropod cell cultures aside from the exceptions discussed in the next section [1,7]. These viruses are commonly referred to as no known arthropod vector (NKV) flaviviruses and can be further divided into bat- and rodent-associated NKV flaviviruses (B-NKV and R-NKV flaviviruses, respectively). The purpose of this article is to provide a review of the classification, host range, geographic distribution, transmissibility, replication kinetics, pathogenesis and molecular biology of NKV flaviviruses.

## 2. Classification

NKV flavivirus is a non-taxonomic designation for flaviviruses that have no apparent arthropod vector. The International Committee on Taxonomy of Viruses (ICTV) recognizes 14 species of NKV flaviviruses [16,17] (Table 1 and Table 2). Of these, eight are bat-associated, and six are rodent-associated. B-NKV flaviviruses are as follows: Bukalasa bat virus (BBV), Carey Island virus (CIV), Dakar bat virus (DBV), Entebbe bat virus (ENTV), Montana myotis leukoencephalitis virus (MMLV), Phnom Penh bat virus (PPBV), Rio Bravo virus (RBV) and Yokose virus (YOKV). Batu Cave virus (BCV) is a subtype of PPBV, and Sokoluk virus (SOKV) is a subtype of ENTV. B-NKV flaviviruses can be further separated into the Rio Bravo virus group (BBV, BCV, CIV, DBV, MMLV, PPBV and RBV) and Entebbe bat virus group (ENTV, SOKV and YOKV). R-NKV flaviviruses are as follows: Apoi virus (APOIV), Cowbone Ridge virus (CRV), Jutiapa virus (JUTV), Modoc virus (MODV), Sal Vieja virus (SVV) and San Perlita virus (SPV), and all belong to a single group known as the Modoc virus group, which clusters phylogenetically with the B-NKV Rio Bravo virus group.

Evidence is accumulating that ENTV group viruses (ENTV, SOKV and YOKV) should not be classified as NKV flaviviruses [37,38,42]. An article published after the Ninth Report of the ICTV describes the isolation of SOKV from wild-caught *Argasidae* spp. ticks in Kyrgyzstan [37]. Additionally, SOKV and ENTV replicate (albeit inefficiently) in *Aedes albopictus* (C6/36) mosquito cells, producing peaks titers of 10^2.7^ and 10^3^ plaque-forming units (pfu)/mL, respectively [42]. The ability of YOKV to infect arthropod cells has not been evaluated, but its 3′ UTR contains sequence motifs more characteristic of those found in mosquito/vertebrate flaviviruses than other NKV flaviviruses [38] (see Section 13). Likewise, the 3′ UTR of ENTV contains motifs that closely resemble those of mosquito/vertebrate flaviviruses [43], while the 3′ UTR of SOKV has not been sequenced. Despite evidence to suggest that ENTV group viruses infect arthropods in nature, they are included in this review, because the ICTV has previously classified them as NKV flaviviruses. 

Tamana bat virus (TABV) is another virus with an ambiguous classification. The virus is not officially recognized by the ICTV as a “species” and instead is considered to be a tentative member of the genus *Flavivirus* [16] or a related unclassified virus. TABV was originally isolated from the insectivorous Parnell’s mustached bat (*Pteronotus parnellii*) and has never been recovered from wild-caught or laboratory-inoculated arthropods [32]. Phylogenetic studies have shown that TABV is more closely related to viruses in the genus *Flavivirus* than to any of the other viruses in the family *Flaviviridae*, but the authors also questioned whether it should be assigned to a new genus [44]. Although TABV is not recognized by the ICTV as a NKV flavivirus, it is discussed in this review because it has many *Flavivirus* characteristics and has never been isolated from arthropods.

**Figure 1:**
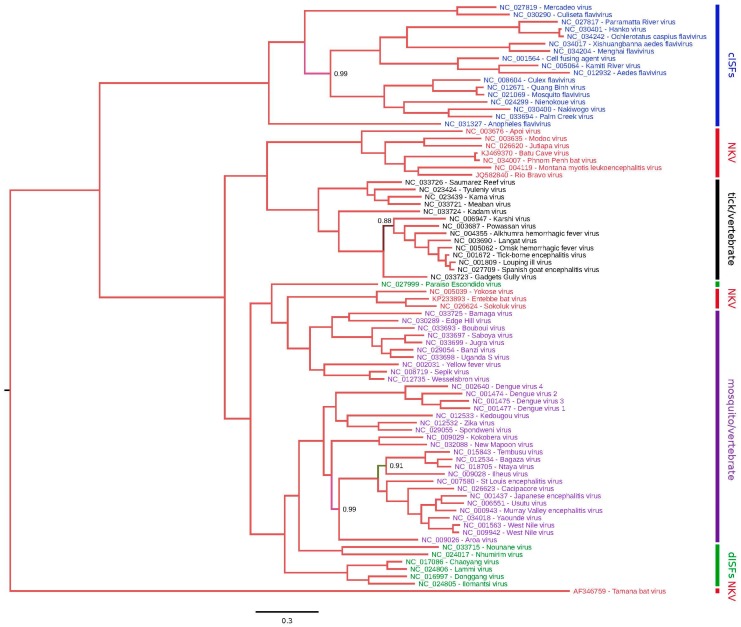
Phylogenetic tree for genus *Flavivirus*. Complete polyprotein amino acid sequences were aligned using MUSCLE [46]. A maximum likelihood phylogenetic tree was estimated using the Bayesian Markov chain Monte Carlo method implemented in MrBayes Version 3.2.3 [45] sampling across the default set of fixed amino acid rate matrices, with ten million generations, discarding the first 25% as burn-in. The figure was produced using FigTree v1.4.2. (http://tree.bio.ed.ac.uk/software/figtree/). The tree is midpoint-rooted; nodes are labelled with posterior probability values if different from 1.00, and poorly-supported branches are also colored differently. Species names are color-coded as follows: classical insect-specific flaviviruses, blue; dual-host affiliated insect-specific flaviviruses, green; NKV flaviviruses, red; mosquito/vertebrate flaviviruses, purple; tick/vertebrate flaviviruses, black. dISFs: dual-host affiliated insect-specific flaviviruses.

**Figure 2:**
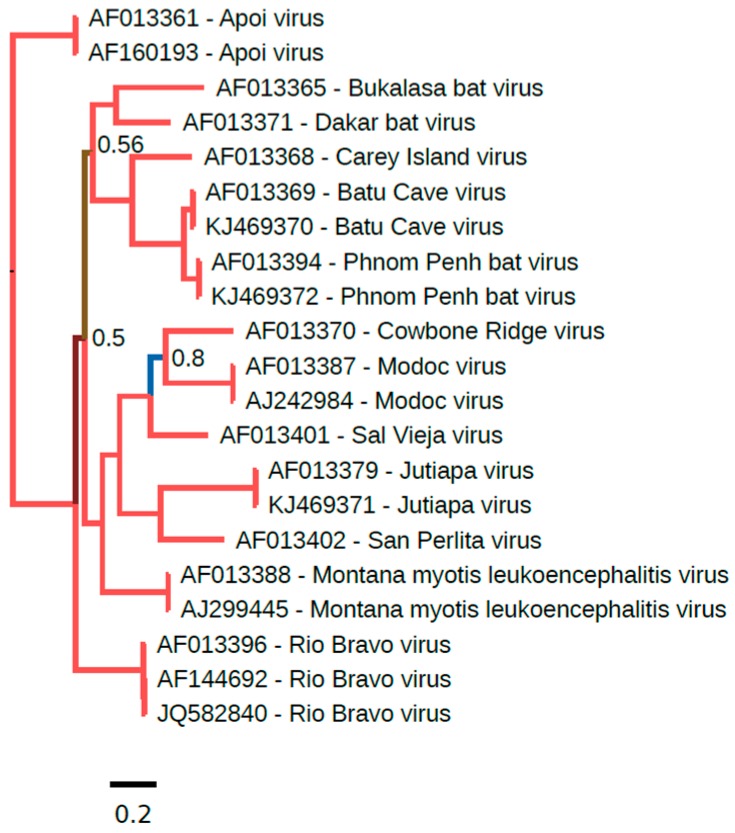
Phylogenetic tree for Rio Bravo virus (RBV)-group partial NS5 sequences. A 1011-nucleotide (nt) region of NS5 corresponding to nt 8952–9962 of JQ582840 (RBV) was used in order to include several additional NKV flaviviruses, for which only partial NS5 sequences are available. The sequences form a gapless nucleotide sequence alignment. A maximum likelihood phylogenetic tree was estimated using the Bayesian Markov chain Monte Carlo method implemented in MrBayes Version 3.2.3 [45] using the general time reversible (GTR) substitution model with the gamma-distributed rate variation across sites and a proportion of invariable sites. Chains were run for one million generations, with the first 25% discarded as burn-in. The figure was produced using FigTree v1.4.2. (http://tree.bio.ed.ac.uk/software/figtree/). Based on the full-genus tree (Figure 1), Apoi virus (APOIV) was selected as an outgroup to root the tree. Nodes are labelled with posterior probability values if different from 1.00, and poorly-supported branches are also colored differently.

The phylogenetic placement of NKV flaviviruses relative to other members of the *Flavivirus* genus was assessed using the Bayesian Markov chain Monte Carlo-based method implemented in MrBayes [45]. Full-length NKV polyprotein amino acid sequences were aligned with polyprotein amino acid sequences from all other genus *Flavivirus* RefSeqs currently available in GenBank using MUSCLE [46] and a phylogenetic tree constructed using MrBayes (Figure 1). YOKV, ENTV and SOKV form one flavivirus clade that clusters with the yellow fever virus/Edge Hill virus groups within the mosquito-borne flavivirus clade, indicating that this group of NKV flaviviruses likely evolved from ancestral arthropod/vertebrate flaviviruses. APOIV, MODV, JUTV, BCV, PPBV, MMLV and RBV form a distinct and completely separate clade of NKV flaviviruses. In the full-polyprotein phylogenetic tree, these NKV flaviviruses cluster more closely with the tick-borne flaviviruses than with the mosquito-borne flaviviruses (Figure 1). It should be noted however that in trees where the alignment is preprocessed with Gblocks [47] to remove poorly-aligned regions, the grouping of NKV flaviviruses with tick-borne flaviviruses is uncertain (low posterior probability; e.g., [11], and an alternative topology places these NKV flaviviruses basal to all arthropod/vertebrate flaviviruses (Gblocks preprocessing was not used here due to the inclusion of the highly divergent TABV sequence in this tree). In E and NS3 trees, these NKV flaviviruses cluster with tick-borne flaviviruses, whereas, in an NS5 tree, these NKV flaviviruses occupy the position basal to tick- and mosquito-borne flaviviruses (Figures S1–S3), indicating a possible recombination event during the evolution of these different flavivirus groups. Curiously, YOKV, SOKV and ENTV also appear to cluster with tick-borne flaviviruses in an NS3 tree. As discussed above, TABV is highly divergent from all other flaviviruses, forming an outgroup to the entire flavivirus phylogeny. Whether flaviviruses originated in arthropods, or in vertebrates, or prior to the evolutionary split between vertebrates and invertebrates remains an open question. Since only partial NS5 sequences are available for a number of NKV flaviviruses in the APOIV-MODV-RBV-MMLV clade, we also constructed a phylogenetic tree for all available partial NS5 sequences in this clade (Figure 2).

**Figure 3:**
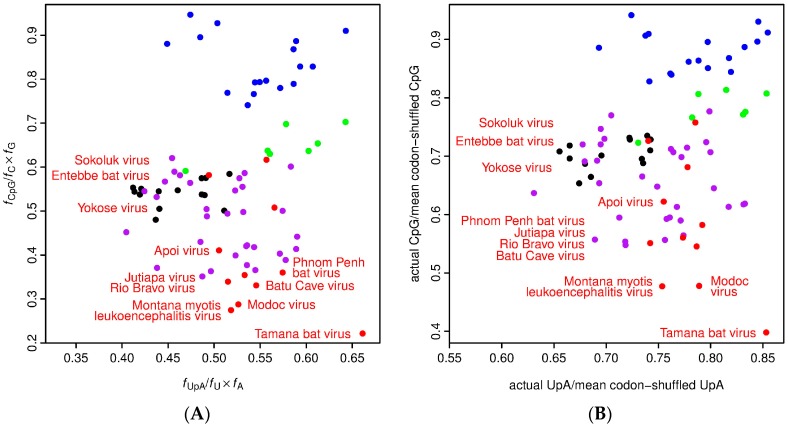
Relative UpA and CpG frequencies in different flavivirus species. UpA and CpG frequencies were calculated in two different ways. (**A**) In each sequence, the numbers of UpA and CpG dinucleotides and A, C, G and U mononucleotides were counted. Dinucleotide frequencies, *f*_XpY_, were expressed relative to their expected frequencies, *f*_X_ × *f*_Y_, in the absence of selection. (**B**) Since codon usage reflects dinucleotide bias, but can also be subject to other selective pressures (e.g., for translational speed or accuracy) that, due to co-evolution of dinucleotide and codon preferences in the host, may lead to the same dinucleotide biases, we also calculated dinucleotide biases independent of codon (and amino acid) usage. To factor out codon and amino acid usage, 1000 shuffled ORF sequences were generated for each virus sequence. In each shuffled sequence, the original amino acid sequence and the original total numbers of each of the 61 codons were maintained, but synonymous codons were randomly shuffled between the different sites where the corresponding amino acid is used in the original sequence. Then, the UpA and CpG frequencies in the original sequence were expressed relative to their mean frequencies in the codon-shuffled sequences. Because codon usage is factored out, the UpA and CpG relative frequencies tend to be less extreme in (**B**) compared to (**A**). Since many sequences lack complete UTRs, for consistency, both analyses of all species were restricted to the polyprotein ORF. Each point represents a single flavivirus sequence. Points and selected species names are color-coded as follows: Classical insect-specific flaviviruses, blue; Dual-host affiliated insect-specific flaviviruses, green; NKV flaviviruses, red; Mosquito/vertebrate flaviviruses, purple; Tick/vertebrate flaviviruses, black. Virus names refer to NKV flaviviruses (red points). GenBank accession numbers are the same as those used in Figure 1.

As mentioned above, there is some evidence that YOKV, ENTV and SOKV are not in fact NKV flaviviruses, but may instead be arboviruses for which a vector has yet to be defined. To further investigate this possibility, we measured UpA and CpG dinucleotide frequencies in the different flavivirus sequences (Figure 3). Vertebrate and arthropod host mRNAs display characteristic under-representation of UpA and CpG (vertebrates) and UpA (arthropods), which, among other things, is thought to be linked to as-yet-uncharacterized host defense mechanisms for recognizing and/or responding to non-self RNA [48,49,50]. Consequently, dinucleotide usage in viruses is subject to selective pressure, and many viruses have evolved dinucleotide usage patterns that at least partly mirror their hosts, so that an analysis of dinucleotide usage may provide an indication of likely host organism [51]. While the RBV/MODV clade of NKV flaviviruses had very low CpG usage (lower on average than arthropod/vertebrate flaviviruses), the CpG usage of YOKV, ENTV and SOKV was similar to that of arthropod/vertebrate flaviviruses, suggesting that they are not specifically adapted to vertebrate hosts (Figure 3). TABV, on the other hand, had the strongest selection against CpG of all of the flavivirus sequences analyzed, consistent with it being a bona fide vertebrate-specific virus.

## 3. Discovery, Geographic Distribution and Natural Host Range

The first B-NKV flavivirus to be discovered was RBV (formerly known as bat salivary gland virus) after its isolation from the salivary glands of a Mexican free-tailed bat (*Tadarida brasiliensis mexicana*) in Rio Bravo, California, in 1954 [33]. Subsequent isolations were made from Mexican free-tailed bats, black mastiff bats (*Molossus rufus*; also known as *Molossus ater*) and a big brown bat (*Eptesicus fuscus*) in Mexico, Trinidad and elsewhere in the United States [24,31,32,52,53]. The first R-NKV flavivirus to be discovered was APOIV after its isolation from pooled spleens from *Apodemus* and *Clethrionomys* spp. mice trapped at the foothills of Mount Apoi, Japan, in 1954 [24]. MODV, the best characterized R-NKV flavivirus, was first isolated four years later. The original isolation was from a deer mouse (*Peromyscus maniculatus*) in Modoc County, California, in 1958 [41], and the virus was later found in Canada and elsewhere in the United States [35,40]. The host ranges and geographic distributions of all NKV flaviviruses (both recognized and tentative) are summarized in Table 1 and Table 2.

## 4. Transmission

Horizontal transmission: NKV flaviviruses are assumed to be maintained in nature by horizontal transmission among reservoir hosts. Compelling evidence of horizontal transmission was first provided by Fairbrother and Yuill [54]. Briefly, MODV-inoculated and non-inoculated deer mice were caged together for six weeks, and contact animals were tested for evidence of infection. Eight of 16 (50%) non-inoculated animals contained MODV-specific antibodies, including one which also contained viral antigen in its lungs. In an earlier experiment, lungs were harvested from MODV-infected mice every week for 10 weeks. Viral antigen was detected in the lungs as early as six weeks post-inoculation (PI) and continued to be detected for the remainder of the observation period. Taken together, the persistence of virus in the lungs and its ability to be transmitted between animals in close contact is suggestive of horizontal transmission by direct contact (possibly from salivary secretions through mutual grooming or biting) or indirect contact (i.e., fomites, aerosols and urine). Some rodent species, such as *Peromyscus* spp. mice, often nest together in large families in the winter, which provides conditions suitable for the horizontal transmission of viruses. 

Davis and colleagues also reported that MODV persists in the lungs of infected deer mice [55]. However, no evidence of horizontal transmission of virus was observed between infected and uninfected mice caged together for four weeks. A likely explanation for the lack of horizontal transmission is that the two groups of animals were housed together for an insufficient amount of time. As noted above, Fairbrother and Yuill [54] demonstrated that at least six weeks is required for MODV to disseminate to the lungs of infected mice. Horizontal transmission of MODV between infected and uninfected golden hamsters (*Mesocricetus auratus*; also known as Syrian hamsters) housed together for four weeks was also inefficient; virus-specific antibodies were detected in only one of 27 (3.7%) contact animals [56]. The potential for MODV to be horizontally transmitted between deer mice by cannibalism has been explored [55]. Uninfected mice were force-fed lung tissues from MODV-infected mice and assayed for virus-specific antibodies four weeks later. Antibodies were not detected in any mice indicating than cannibalism does not have an important role in the maintenance of NKV flaviviruses in nature. However, we cannot dismiss the possibility that the length of the study was not sufficient to detect a serological response in the mice. The potential for NKV flaviviruses to be sexually transmitted among reservoir hosts has not been explored.

Laboratory experiments have not directly evaluated the potential for B-NKV flaviviruses to be horizontally transmitted between bats in close contact. However, B-NKV flaviviruses have been isolated from the lungs, salivary glands and saliva of naturally- and experimentally-infected bats indicating that horizontal transmission could occur through aerosol exposure or some form of salivary contact. RBV and TABV have been isolated from the salivary glands and saliva of naturally-infected bats [31,32], and ENTV was recovered from the salivary glands and lungs of bats with naturally-acquired infections [27,28]. MMLV was originally isolated from a laboratory mouse bitten by a naturally-infected little brown bat (Myotis lucifugus) and later isolated from the saliva and various tissues of other little brown bats [29]. In another study, direct transmission of TABV occurred after infected saliva from greater spear-nosed bats (*Phyllostomus hastatus*) was subcutaneously inoculated into recipient bats of the same species [32]. Bats often congregate in large numbers in poorly-ventilated caves, which provides conditions conducive for horizontal virus transmission. The social behavior of bats may also facilitate horizontal virus transmission. 

Vertical transmission: A limited number of studies has investigated the vertical transmission potential of R-NKV flaviviruses [55,56], and none have directly assessed the ability of their bat-associated counterparts to be transmitted by this mechanism. Nonetheless, it is assumed that vertical transmission does not play an important role in NKV flavivirus maintenance in nature. In one study, MODV could not be isolated from any offspring produced by deer mice that had been infected 17–54 days prior to giving birth [55]. Antibodies to MODV were detected in 11 of 14 (79%) offspring 2–7 days post-birth, but were not detected at any other times, leading the authors to conclude that they were maternally derived. The potential for suckling mice to become infected by the consumption of lactating fluids was also explored. Adult females that had given birth were inoculated with MODV in their first postnatal week and returned to their offspring. MODV was not isolated from any progeny, but four of 19 (21%) contained virus-specific antibodies at 38 days post-birth, suggesting that limited vertical transmission had occurred. In this regard, MODV has been isolated from the mammary glands of naturally- and experimentally-infected mice [41,55]. Female golden hamsters infected with MODV 7–11 weeks before they bred failed to transmit virus to any of their progeny [56]. The authors also reported that all progeny from hamsters infected with virus 7–9 days before they littered were stillborn or displayed signs of encephalitis and died shortly after birth [56]. Taken together, the above findings indicate that vertical transmission is an inefficient mechanism for the maintenance of NKV flaviviruses in nature.

## 5. Replication Kinetics, Persistence and Pathogenesis of B-NKV Flaviviruses in Bats

Acute infection: Information on the replication kinetics and tissue tropisms of B-NKV flaviviruses in bats during acute infection is limited [32,57,58]. In one study, 19 greater spear-nosed bats (*Phyllostomus hastatus*) were experimentally-inoculated with TABV [32]. Virus was isolated from sera, spleens, salivary glands and saliva as early as 2, 6, 6 and 7 days PI, respectively, but was not recovered from brain, liver, pancreas or kidney. Antibodies to TABV were first detected by hemagglutinin-inhibition and neutralization tests at six days PI and remained detectable until the final bat was euthanized at 60 days PI. Signs of illness were not observed in any bats. Three Seba’s short-tailed bats (*Carollia perspicillata*) and one Jamaican fruit bat (*Artibeus jamaicensis*) were also inoculated with TABV. Virus was not recovered from the saliva of any bats at seven or 14 days PI, nor was it recovered from any sera at 14 days PI, but hemagglutinin-inhibition antibodies were detected in both species at this time. All bats appeared healthy for the duration of the experiment. 

The replicative potential and tissue tropisms of YOKV in Leschenault’s rousette fruit bats (*Rousettus leschenaultii*) inoculated by the intraperitoneal (i.p.) route have been assessed [58]. Viral RNA was not detected in any organs (brain, heart, kidney, liver, lung and spleen) or any other samples (sera, urine and feces), except for one liver sample. Neutralizing antibodies to YOKV were not identified in any bats, and none developed clinical signs. The authors concluded that Leschenault’s rousette bats do not support efficient replication of YOKV and are therefore unlikely to be reservoir hosts. In another study, no evidence of ENTV replication was observed in experimentally-inoculated frugivorous and insectivorous bats [57].

Persistence: B-NKV flaviviruses persistently infect their chiropteran hosts. Mexican free-tailed bats with naturally-acquired RBV infections were held in the laboratory for extended amounts of time and periodically tested for virus [31]. RBV was isolated from saliva as long as 282 days post-capture and from salivary glands (but not brains, lungs or kidneys) upon euthanasia at 682 days post-capture. In a similar study, RBV was isolated from saliva collected from a naturally-infected Mexican free-tailed bat up until the time of euthanasia at 309 days post-capture [34]. Virus was also recovered from the salivary glands, but not the brain, lung, heart, kidney, intestine, spleen, pancreas, liver or reproductive organs. RBV was isolated from the salivary glands of 24 of 1075 (2.2%) naturally-infected Mexican free-tailed bats, but it is not known if any had chronic infections because they had been infected for an unknown amount of time [31]. Virus was also recovered from the lungs of one bat in this study, but all other organs (brains, kidneys and mammary glands) were negative. To our knowledge, saliva from RBV-infected bats has never been tested for antibodies to RBV. Taken together, the above findings indicate that the B-NKV flaviviruses persist in the salivary glands and saliva of their hosts. 

Pathogenesis: B-NKV flaviviruses presumably establish asymptomatic infections in their chiropteran hosts. As already noted, signs of illness were not observed in any bats challenged with TABV or YOKV [32,57,58]. In another study, RBV was isolated from 33 naturally-infected Mexican free-tailed bats of which 26 were asymptomatic and seven were dying or dead [31]. However, the authors concluded that RBV did not cause the fatalities because it was recovered from asymptomatic and dying/dead bats at similar frequencies. RBV was also isolated from three of 44 (6.8%) weak Mexican free-tailed bats that died soon after capture and from one of two (50%) others that survived captivity [34]. The prevalence of RBV in healthy bats in the same study area was not determined because bats that appeared unwell were specifically targeted. 

## 6. Replication Kinetics, Persistence and Pathogenesis of R-NKV Flaviviruses in Rodents

Acute infection: R-NKV flaviviruses disseminate to a broad range of organs and body fluids of their rodent hosts during acute infection. Davis and colleagues inoculated deer mice with MODV by the intranasal (i.n.) route and recovered virus from all organs and body fluids tested [55]. Virus was isolated from lungs and throat swabs of mice sacrificed immediately after inoculation and first recovered from spleen, salivary glands, kidney, blood and bone marrow at two days PI, lymph nodes and spinal cord at three days PI, heart and urine at four days PI and brain and liver at five days PI. Virus titers were highest (>10^6^ pfu/g) in spleens followed by salivary glands, lungs and lymph nodes. Titers peaked in lungs at two days PI and in spleens, salivary glands and lymph nodes at six days PI. Viremias were detectable from 1–4 days PI and never exceeded 10^3.4^ pfu/mL. The detection of MODV in the lungs immediately after inoculation is in contrast to the findings of Fairbrother and Yuill [54], who reported that at least six weeks is required for the virus to disseminate to the lungs. It is possible that these different findings are due to the inoculation methods used. Davis and colleagues infected mice by the i.n. route, which permits almost instantaneous exposure of the lungs to the virus, while Fairbrother and Yuill [54] performed i.p. inoculations. 

In another study, low viremias persisted for four days in deer mice infected with MODV by the i.p. route [54]. Antibody titers were first detected by complement-fixation test at eight days PI and peaked at 13–20 days PI. Golden hamsters infected with MODV by the i.n. route had detectable viremias at 2–6 days PI [56]. Viremias peaked (10^6.2^ pfu/mL) at four days PI. Neutralizing and hemagglutinin-inhibition antibodies were first detected in all animals at seven days PI. Complement-fixation antibodies were first detected in all animals at 14 days PI. Golden hamsters inoculated with MODV by the subcutaneous (s.c.) route had hemagglutinin-inhibition antibody titers ranging from 320–1280 at 10 days PI [59].

Persistence: Information on the ability of R-NKV flaviviruses to persistently infect rodents has primarily been obtained from studies performed with MODV. Johnson [60] demonstrated that hamsters infected with MODV by the intramuscular (i.m.) route excrete virus in their urine for at least 153 days. Virus was also recovered from kidneys as far as 424 days PI. Another study revealed that MODV persists in the urine and kidneys of golden hamsters for up to five and eight months, respectively after infection by s.c. inoculation [59]. MODV also chronically infects golden hamsters and deer mice injected by the i.n. route despite the presence of neutralizing antibodies [40,41]. MODV was not isolated from urine, feces or oral swabs collected from deer mice over a 63-day period after infection by the intraperitoneal (i.p.) route [54]. The authors speculated that the lack of virus shedding in their study [54], which is in contrast to the other aforementioned studies [40,41,48,49], was due to differences in the inoculation routes or virus strains used. 

Pathogenesis: Like their bat-associated counterparts, R-NKV flaviviruses presumably do not usually cause overt disease in their natural reservoir hosts. However, illness has been observed in rodents infected in the laboratory. Fatal encephalitis occurred in two of 16 (12.5%) golden hamsters infected with MODV by the s.c. route [59]. Viral antigen was detected in the neurons of the brainstem and spinal cord during acute infection. Mortality rates as high as 50% were reported for golden hamsters inoculated with MODV by the i.p. or i.n. routes [61]. It is important to note that hamsters have not been implicated as natural reservoir hosts of NKV flaviviruses. Suckling deer mice infected with MODV by the intracranial (i.c.) route displayed signs of illness at 9–16 days PI [60]. A sick mouse is the likely source of an MODV infection acquired by a young boy [62,63].

## 7. Human Disease

NKV flaviviruses are not usually associated with human disease, although several cases have occurred (Table 1 and Table 2). DBV was responsible for two natural cases of febrile illness in humans in Nigeria [24,64]. Isolates were recovered from both patients, and to the best of our knowledge, NKV flaviviruses have not been isolated from any other naturally-infected humans. One natural and seven laboratory-acquired cases of RBV have been documented with symptoms ranging from mild febrile illness to lymphadenopathy, orchitis or oophoritis, as well as central nervous system involvement [24,65,66]. Laboratory exposure is suspected to have occurred by the aerosol route. Two R-NKV flaviviruses, APOIV and MODV, have been associated with human disease. APOIV was responsible for an accidental laboratory infection characterized by fever, headache, myalgia, arthralgia, encephalitis and sequela (paralysis in legs) [24]. MODV was implicated as the cause of aseptic meningitis in a male child who had handled a sick mouse a few days before symptom onset [62,63]. 

## 8. Disease in Other Vertebrates

NKV flaviviruses have been demonstrated to cause disease in non-human primates (NHPs) in the laboratory [32,65]. A white-faced capuchin monkey (*Cebus nigrovittatus*) infected with TABV by the s.c. route displayed signs of illness (i.e., lying in a hunched position and refusal of food and water) at 10 days PI [32]. Virus was isolated from its saliva as early as four days PI. The monkey, although slowly recovering, was still symptomatic at the time of euthanasia (21 days PI). Rhesus monkeys (*Macaca mulatta*) infected with RBV by i.n. inoculation became febrile at 6–7 days PI [65]. Monkeys inoculated by the intravenous and i.m. routes developed biphasic fevers at 5–6 and 9–11 days PI. All monkeys became viremic by 1–2 days PI and developed neutralizing antibodies. There is no evidence to suggest that NKV flaviviruses infect NHPs in nature. 

B-NKV flaviviruses cause severe illness in mice and rats infected under laboratory conditions [29,31,32,34,67,68]. Fatal illness occurred in infant mice inoculated with TABV by the i.c. and i.p. routes [32]. An infant rat challenged with TABV by the i.c. route also succumbed to infection. Three-week-old mice inoculated with RBV by the i.c. route exhibited signs of paralysis [31]. ENTV causes illness in adult mice infected by the i.c. route and in infant mice infected by the i.c. and i.p. routes [27,69]. There are no reports of naturally-acquired B-NKV flavivirus infections in rodents. 

The ability of NKV flaviviruses to infect chickens and rabbits in the laboratory has been evaluated [24]. Chick embryos (eight days old) inoculated with MMLV by the amniotic sac route usually died at six days PI. Chick embryos (five days old) infected with RBV or MODV by the yolk sac route sometimes succumbed to infection at 4–5 and 4–9 days PI, respectively. Viremia was detected in chicks (one day old) infected with either RBV or MODV by the i.m. route, but none of the infections were fatal. DBV or MMLV were not pathogenic for rabbits inoculated by the i.c. route. There is no evidence to indicate that NKV flaviviruses infect rabbits [70] or birds in nature. 

## 9. Serological Cross-Reactivity

In a classic study by Varelas-Wesley and Calisher, the antigenic relationships between every recognized and tentative NKV flavivirus, except for YOKV, were characterized by complement-fixation test with most viruses also examined by plaque reduction neutralization test (PRNT) [42]. In the complement-fixation tests, antibodies to three B-NKV flaviviruses (BBV, MMLV and TABV) reacted significantly only with homologous antigens. PPBV and its subtype BCV were indistinguishable from one another, unlike ENTV and its subtype SOKV, which could be distinguished. Antibodies to RBV primarily cross-reacted with APOIV and SPV antigens. Antibodies to CIV and DBV either failed to react or weakly reacted with heterologous antigens. Antibodies to all R-NKV flaviviruses, except APOIV, were moderately to highly cross-reactive to at least one type of heterologous antigen. Significant cross-reactivity did not occur when APOIV antiserum was used. In the PRNTs, antibodies to most B-NKV flaviviruses significantly neutralized only homologous virus while all R-NKV flaviviruses, except for APOIV, cross-neutralized most or all other R-NKV flaviviruses. Taken together, these data indicate that antigenic cross-reactivity occurs between some NKV flaviviruses. These findings are in agreement with an earlier study in which mice immunized by the i.m. route with MODV were resistant to i.c. challenge with RBV [41]. In the reciprocal experiment, RBV-immunized mice were partially resistant to MODV challenge. Antigenic cross-reactivity also occurs between some arthropod/vertebrate and NKV flaviviruses. For example, antibodies to RBV were able to neutralize Aroa virus (a mosquito/vertebrate flavivirus) [42]. Neutralization also occurred in the reciprocal experiment.

## 10. Seroprevalence

There is limited information on the seroprevalence of NKV flaviviruses in humans and vertebrate animals. Additionally, most data were obtained in serological investigations performed decades ago using traditional serologic assays (i.e., hemagglutinin-inhibition and neutralization tests), which are far less specific than the PRNT commonly used nowadays for flavivirus serodiagnosis. In one early study, RBV-reactive antibodies were detected by hemagglutinin-inhibition assay in 49 of 169 (30.0%) humans and 125 of 887 (14.1%) bats in Trinidad in 1972–1974 [32]. The authors also detected TABV-reactive antibodies in 21 of 172 (12.2%) humans and 72 of 850 (8.5%) bats. Due to the limited specificity of the hemagglutinin-inhibition assay, it is not possible to determine whether some (or all) of the infections were caused by antigenically similar flaviviruses instead of the aforementioned NKV flaviviruses. RBV- and TABV-reactive antibodies were also detected by hemagglutinin-inhibition assay in bats in Trinidad in 2006–2008 [71]. In another study, DBV-reactive antibodies were detected by the hemagglutinin-inhibition assay in nine of 300 (3.0%) humans and 90 of 165 (54.5%) bats in Senegal in the 1960s [72]. MODV-reactive antibodies were detected by the neutralization test in three of 50 (6.0%) humans, 13 of 109 (11.9%) deer mice, three of 35 (8.6%) least chipmunks (*Eutamias minimus*) and one of 38 (2.6%) red squirrels (*Tamiasciurus hudsonicus*) in Canada in 1976 [40]. A titer as low as 1:10 was considered a positive result, and therefore, another flavivirus(es) could have easily been responsible for some (or all) of the infections detected. Blacktail jackrabbits (*Lepus californicus*) in California in 1971–1974 were tested by the hemagglutinin-inhibition test for antibodies to multiple viruses [70]. None had RBV- or MODV-reactive antibodies.

Few studies have used the PRNT to estimate the seroprevalence of NKV flaviviruses in nature. House mice (*Mus musculus*) and black rats (*Rattus rattus*) in Mexico in 2011–2012 were tested by PRNT for antibodies to seven flaviviruses, including two R-NKV flaviviruses (MODV and APOIV) [73]. Thirteen of 86 (15.1%) house mice and 48 of 75 (64.0%) black rats were PRNT-positive for flavivirus cross-reactive antigen, but only two rats were considered to have antibodies to MODV or a MODV-like virus. Bats trapped in Guatemala in 1983–1984 were tested by PRNT for antibodies to multiple viruses including two flaviviruses: RBV and St. Louis encephalitis virus (a mosquito/vertebrate virus) [74]. Fifty of 271 (18.5%) bats had antibodies that neutralized RBV. Because end-point titers were not determined and only one other flavivirus was included in the analysis, it is unclear whether RBV or an antigenically similar flavivirus was the etiological agent.

## 11. In Vitro Replication Kinetics

Vertebrate cells: Human, monkey, rodent and avian cell lines support the in vitro replication of NKV flaviviruses. The most comprehensive analysis was performed using RBV, which was shown to replicate in monkey (Vero, LLC-MK2), rodent (BHK-21, L) and human (HeLa, Fogh and Lund (FL) amnion) cell lines, as well as human embryo skin cells and primary chick embryo fibroblasts [75]. Replication was most efficient in BHK-21 and Vero cells; the virus reached peak titers of 10^7.9^ and 10^6.9^ per mL, respectively (titers determined by i.c. inoculation of suckling mice and expressed as lethal dose 50%). Replication in chick embryo fibroblasts was modest; the virus reached a peak titer of 10^4.0^ per mL. L, HeLa and FL amnion cells only supported RBV replication when they were inoculated with high concentrations of virus. Another study revealed that RBV can cause plaques in Vero cells, LLC-MK2 cells and duck embryo cultures [24]. Bat lung and primary duck embryo cells support the replication of RBV at 36 °C, but not 42 °C [76]. Vero, LLC-MK2 and BHK-21 cells have also been shown to support the replication of other B-NKV flaviviruses, including DBV, ENTV and MMLV [24].

The best studied R-NKV flaviviruses in terms of their abilities to replicate in vertebrate cell lines are MODV, APOIV and CRV [24,63]. Efficient replication of MODV occurs in Vero cells, LLC-MK2 cells and duck embryo primary cultures; the virus reaches peak titers of 10^8.0^, 10^7.7^ and 10^7.0^ pfu/mL, respectively [24]. MODV plaques in Vero cells; plaque diameters ranged from 0.5–2 mm at 6–7 days PI [63]. APOIV replicates in BHK-21, Vero, FL and HeLa cells [24]. APOIV plaques in chick embryo cells; a peak titer of 10^8.4^ pfu/mL occurs at four days PI. CRV plaques in Vero, LLC-MK2 and L cells, but not duck embryo cells.

Arthropod cells: As noted earlier, ENTV and SOKV replicate (albeit inefficiently) in *Aedes albopictus* (C6/36) cells [42]. All other attempts to infect arthropod cells with NKV flaviviruses have proven unsuccessful. The most comprehensive in vitro host range studies were performed using MODV and RBV; both viruses failed to replicate in *Culex tarsalis* [77], *Aedes dorsalis* [78] and *Aedes albopictus* [42] mosquito cell lines. MODV also lacks the capacity to replicate in *Dermacentor variabilis* and *D. parumapertus* tick cell lines [79]. 

## 12. Genome Sequencing

Complete genomic sequences are available for three B-NKV flaviviruses: MMLV, RBV and YOKV [38,80,81]. The genomes range in length from 10,690 to 10,857 nt and contain 5′ and 3′ UTRs of 108–150 nt and 429–486 nt, respectively (Table 3). The genomes of ENTV and TABV have also been fully sequenced except for the distal 3′ ends of their respective 3′ UTRs [43,44]. Complete polyprotein ORF sequences are available for BCV, PPBV and SOKV, and partial NS3 and/or NS5 gene sequences are available for BBV, CIV and DBV. The only R-NKV flavivirus for which the genome has been fully sequenced is MODV (Table 4). This genome consists of 10,600 nt and contains 5′ and 3′ UTRs of 109 and 366 nt, respectively [82]. Complete polyprotein ORF sequences are available for two other R-NKV flaviviruses (APOIV and JUTV), and partial NS3 and/or NS5 gene sequences are available for all others (CRV, SVV and SPV).

## 13. Flavivirus 3′ UTRs: Insights into Host Specificity 

The sequence and organization of the flavivirus 3′ UTR are dependent upon host specificity. Two highly conserved primary sequence elements present in the 3′ UTRs of mosquito/vertebrate flaviviruses are conserved sequence 1 (CS1) and conserved sequence 2 (CS2) [1,2,83,84]. The 3′ UTR of YOKV also contains CS1 and CS2 sequences, leading to the speculation that YOKV utilizes a mosquito vector in nature [38]. ENTV contains a CS2 sequence, but the existing partial 3′ UTR sequence appears to terminate 5′ of the expected position of CS1. In contrast to mosquito/vertebrate flaviviruses, MMLV, MODV, RBV and APOIV contain CS2, but not CS1 sequences [81]. CS2 is part of a larger RNA “dumbbell” (DB) secondary structure that is present in the 3′ UTRs of all mosquito/vertebrate and NKV flaviviruses, often in tandem copies, but not in tick/vertebrate flaviviruses or classical insect-specific flaviviruses [85,86].

In common with mosquito/vertebrate flaviviruses, YOKV and ENTV contain an RNA sequence that folds into a pseudoknotted structure that resists host exoribonuclease XRN1-mediated 5′ to 3′ degradation of the viral genome during infection, leading to the accumulation of a subgenome-sized RNA, subgenomic flavivirus RNA (sfRNA), corresponding to the 3′ terminal region of the virus genome [87,88,89]. Many mosquito/vertebrate flaviviruses contain tandem copies of the RNA structure, but in ENTV and YOKV, as in YFV, it is present as a single copy. This structure appears to be absent from the 3′ UTRs of MMLV, MODV, RBV and APOIV.

Four structural regions have been identified in the 3′ UTRs of MMLV, MODV, RBV and APOIV by comparative analysis [81], including the aforementioned DB (region II; duplicated in APOIV), the 3′-terminal sHP-3′SL (region IV; see below), a Y-shaped structure with conserved AUUGGC and (U/G)(U/G)UU loop motifs (region III) and a different Y-shaped structure (region I; absent in APOIV). The region I and region III structures are similar to structures present in the 3′ UTRs of tick-borne flaviviruses, designated Y-shape stem-loop (Y-SL) and AU containing stem-loop (AU-SL), respectively [85].

The 3′-terminal region of all flavivirus 3′ UTRs is predicted to fold into a long stem-loop structure preceded by a small hairpin stem-loop (sHP-3′SL) that, together with a Y-shaped stem-loop structure (SLA) in the 5′ UTRs, is essential for genome replication (reviewed in [90]. In mosquito/vertebrate flaviviruses, the sHP overlaps the CS1 sequence. Nucleotides 1–4 of the (normally) 7-nt terminal loop of the 3′SL, together with the 5′ flanking nucleotide, comprise a CACAG pentanucleotide that is highly conserved in both tick/vertebrate and mosquito/vertebrate flaviviruses (but is CACCG in Murray Valley encephalitis virus and CGCCG in YOKV; it is also CACCG in classical insect-specific flaviviruses). Curiously, this pentanucleotide is replaced with a C(C/U)(C/U)AG sequence in MMLV, MODV, RBV and APOIV [81]. Flavivirus 3′ UTRs also contain cyclization sequences that base pair with sequences in the 5′ UTRs and/or capsid coding regions, leading to non-covalent circularization of the genome. The SLA element in the 5′ UTR binds the viral polymerase, NS5, and genome cyclization is thought to allow delivery of the polymerase to the 3′ end of the genome to initiate minus-strand synthesis (reviewed in [90,91]). The formation of the cyclization base-pairings normally competes with the aforementioned 3′ UTR structures, thus potentially acting as a switch between competing roles of the genomic RNA in translation, replication and encapsidation.

## 14. Predicted Polyprotein Cleavage Sites

The NKV flavivirus polyprotein cleavage sites were predicted by alignment to known cleavage sites in arthropod/vertebrate flaviviruses (Table 5), aided by SignalP-4.1 [92] predictions for the signalase cleavage sites. For the most part, these sites conform to the rules established for arthropod/vertebrate flaviviruses, although there are some exceptions. The most notable example is TABV; due to high sequence divergence from other flaviviruses, several of the TABV cleavage sites remain uncertain (see also [44]). 

Studies performed with arthropod/vertebrate flaviviruses have revealed that a host signal peptidase mediates cleavage between C/prM, prM/E, E/NS1 and 2K/NS4B and that these junctions typically conform to predicted signalase cleavage sites [93]. Similar sites were identified at the predicted C/prM, prM/E, E/NS1 and 2K/NS4B junctions of NKV flavivirus genomes, with the exception of the TABV genome for which the C/prM and 2K/NS4B sites are not well conserved. The NS1/NS2A cleavage site of arthropod/vertebrate flaviviruses is signalase-like with respect to the ′−1, −3′ rule, but an upstream hydrophobic domain is absent. In the arthropod/vertebrate flaviviruses, NS1/NS2A cleavage usually occurs after a Val-X-Ala site. The same motif was identified at the predicted NS1/NS2A cleavage site of every NKV flavivirus, although the potential cleavage site identified in TABV was less certain as it did not align with the cleavage site in other flavivirus sequences. In arthropod/vertebrate flaviviruses, the cellular protease furin cleaves prM to generate the mature form of the protein [93,94]. Furin normally cleaves after the motif Arg-X-Lys/Arg-Arg, but cleavage can also occur after Arg-X-X-Arg [95]. Consistently, the predicted pr/M junction of every NKV flavivirus is preceded by Arg-X-Lys-Arg or Arg-X-Arg-Arg.

The virion C/anchor, NS2A/NS2B, NS2B/NS3, NS3/NS4A, NS4A/2K and NS4B/NS5 junctions of arthropod/vertebrate flaviviruses are cleaved by the viral NS2B/NS3 serine protease, which normally cleaves after two basic amino acid residues (Lys-Arg, Arg-Arg or Arg-Lys) or sometimes after Gln-Arg at the P_2_ and P_1_ positions, followed by a small amino acid (Gly, Ala or Ser) at the P’_1_ position [93,96,97], although there are exceptions such as the dengue virus NS4A/2K site, where cleavage takes place between Gln-Arg and Thr. Two basic amino acids were usually identified at the corresponding predicted cleavage sites of most NKV flaviviruses. The most notable exception is TABV, which lacks a basic amino acid residue at the P_2_ position of most of the aforementioned junctions. There are several other instances where a basic amino acid residue was not present at the P_2_ position; for example, all three ENTV group viruses have Thr-Arg at the P_2_ and P_1_ positions of the predicted NS3/NS4A junction. Similar to dengue virus, in most of the NKV flaviviruses, cleavage at the NS4A/2K junction was predicted to occur between Gln-Arg and Thr. Thr was also the P’_1_ residue at the predicted NS3/NS4A cleavage site for BCV and PPBV. However, these are predictions, and experimental data (e.g., amino acid sequencing) are needed to conclusively identify the cleavage sites in the polyproteins of NKV flaviviruses. This is particularly the case for TABV where sequence alignments with flavivirus species for which cleavage sites have been determined experimentally are often poor. 

## 15. Biochemical, Biophysical and Molecular Studies

A limited number of biochemical, biophysical and molecular studies has been performed using NKV flaviviruses. One of the earliest studies was performed by Hendricks and colleagues and revealed that the density and virion size of RBV (1.18 g/mL in sucrose gradients and 42 nm in diameter, respectively) are similar to those of arthropod/vertebrate flaviviruses [98]. ENTV and CRV are slightly smaller with average virion diameters of 35–38 nm [39,67]. In another early study, the effects of temperature on the stability of MODV were examined [63]. The virus was remarkably stable when incubated for 72 h in phosphate-buffered saline (pH 7.4) at 7 °C and 22 °C, but incubation at 37 °C resulted in a 10^2.0^–10^3.0^ reduction in viral titer. The effect of pH on RBV stability was assessed by incubating the virus for 2 h in phosphate-buffered saline (PBS) containing fetal calf serum [75]. The optimal pH was 7.6 with no loss in viral titer relative to the starting amount. Viral titers decreased 1.6–6.3-fold at pHs of 7.4–7.2, respectively, and 100–250-fold at pHs of 6.8 and 6.6, respectively.

More recently, the methyltransferase (MTase) domains of MODV and YOKV were examined by protein crystallography [99,100,101]. This domain is located in the amino-terminal region of NS5 and plays a critical role in the capping of the viral genome. Preliminary X-ray diffraction studies on the MTase of YOKV resulted in a structure with 2.7 Å resolution [100]. In a subsequent study, a shorter construct was used, resulting in the production of higher quality crystals, leading to a structure with 1.7 Å resolution [99]. A comparative analysis of the crystal structures of the MTases of YOKV and representative mosquito/vertebrate and tick/vertebrate flaviviruses revealed that they are well conserved. The MTase of MODV also shares many structural characteristics with those of arthropod/vertebrate flaviviruses [101].

Chimeric viruses have been generated between NKV and mosquito/vertebrate flaviviruses [102,103,104,105]. Five chimeric viruses were produced, and each contains genetic elements from MODV, as well as YFV, WNV or dengue virus type-4 (DENV-4). The first chimeric virus was produced by replacing the prM-E genes of YFV with the corresponding genes of MODV [103]. The virus replicated in C6/36 cells, indicating that the inability of NKV flaviviruses to infect mosquito cells is not mediated by the viral envelope, but by a post-entry event [102]. Substitution of the prM-E genes of DENV-4 with those of MODV also resulted in a chimeric virus capable of C6/36 cell replication. Additional chimeric viruses were constructed by replacing the conserved pentanucleotide sequence (CPS) or variable region (VR) of the 3′ UTR of DENV-4 with the corresponding regions of MODV [105]. Both viruses could infect C6/36 cells and adult mosquitoes, indicating that the CPS and VR of mosquito/vertebrate flaviviruses are not required for mosquito infectivity. Another chimeric virus, the first to be constructed using a NKV flavivirus as the backbone, was generated by exchanging the prM-E genes of MODV with those WNV [104]. The virus replicated in Vero and BHK-21 cells, but not C6/36 cells, suggesting that sequence elements outside of the prM-E region dictate the vertebrate-restricted host range of MODV. 

## 16. Concluding Remarks

NKV flaviviruses are poorly characterized compared to their arthropod/vertebrate-infecting counterparts, largely because their impact on human and veterinary animal health is minimal. As already explained, no more than four cases of human disease have occurred due to NKV flavivirus exposure outside of the laboratory, and there have been no reports of naturally-occurring disease in livestock or poultry. It is also highly likely that NKV flaviviruses are greatly under-sampled relative to arthropod/vertebrate flaviviruses because virus surveillance and discovery are often designed to identify viruses of medical and veterinary significance. 

Although NKV flaviviruses are not regarded as a top research priority, there are several important reasons why they should not be neglected by the scientific community. Comparative studies on NKV and arthropod/vertebrate flaviviruses will provide unique insight into why some flaviviruses possess the capacity to cycle between disparate hosts and cause devastating disease in humans and other vertebrates while others do not. This issue can be addressed by the generation and subsequent characterization of additional chimeric viruses of arthropod/vertebrate and NKV flaviviruses in order to identify the genetic elements that preclude mosquito infectivity. This information could assist in the development of live-attenuated flavivirus vaccines that lack the capacity to infect mosquitoes and initiate unwanted transmission cycles. 

NKV flavivirus research could also provide information that will assist in the development of chemoprophylactic and chemotherapeutic strategies against medically-important arthropod/vertebrate flaviviruses that are difficult to work with in the laboratory. Leyssen and colleagues demonstrated that MODV is equally susceptible to select antiviral agents as YFV (a biosafety level-3 agent) and DENV-2 (which replicates poorly in most strains of mice) [106]. The authors also reported that severe combined immunodeficiency (SCID) mice pretreated with inosinic-polycytidilic acid (poly IC; an interferon inducer) are resistant to MODV-induced morbidity and mortality. Therefore, MODV and poly IC-treated SCID mice, when used in tandem, potentially provide an effective model for the identification and characterization of antiviral agents directed against arthropod/vertebrate flaviviruses. In a follow-up study, the same research group provided evidence that SCID mice infected with MMLV can be used as a model to study flavivirus encephalitis [68]. MODV-infected hamsters and mice provide an effective surrogate for the study of central nervous system manifestations (i.e., encephalitis, flaccid paralysis, poliomyelitis-like illness, neuronal dysfunction and neurological sequelae) that are characteristic of JEV and WNV infections (both are BSL-3 agents) [61,107]. 

To conclude, NKV flavivirus research is important because it offers unique insight into the transmission, tropisms, pathogenesis, prevention and treatment of medically-important arthropod/vertebrate flaviviruses. It is also important that future studies focus on the discovery and characterization of unrecognized NKV flaviviruses, because it is very likely that many more occur in nature. These studies would provide information that would greatly increase our understanding of flavivirus evolution and emergence. 

## Figures and Tables

**Table 1:** Geographic distribution, natural host range and clinical manifestations of bat-associated no known vector (NKV) flaviviruses.

Virus	Human Disease	Year of First Isolation	Geographic Distribution	Natural Host Range ^a^	References
Batu Cave virus (BCV) ^b^	No	1971	Malaysia	*Cynopterus brachyotis* (lesser short-nose fruit bat), *Eonycteris spelaea* (dawn bat)	[18,19]
Bukalasa bat virus (BBV)	No	1963	Senegal, Uganda	*Chaerephon pumila* (little free-tailed bat), *Tadarida (Mops) condylurus* (Angolan free-tailed bat)	[20,21,22,23]
Carey Island virus (CIV)	No	1970	Malaysia	*Cynopterus brachyotis* (lesser short-nosed fruit bat), *Macroglossus lagochilus* (lesser long-tongued fruit bat)	[24]
Dakar bat virus (DBV)	Yes (fever)	1962	Central African Republic, Madagascar, Senegal, Nigeria, Uganda	*Chaerephon pumilus* (little free-tailed bat), *Scotophilus* *nigrita* (giant house bat), *Tadarida (Mops) condylurus* (Angolan free-tailed bat), *Taphozous perforatus* (Egyptian tomb bat), *Homo sapiens* (human)	[20,21,23,24,25,26]
Entebbe bat virus (ENTV) ^c^	No	1957	Uganda	*Chaerephon (Tadarida) pumilus* (little free-tailed bat) ^d^	[21,27,28]
Montana myotis leukoencephalitis virus (MMLV)	No	1958	United States	*Myotis lucifugus* (little brown bat)	[29]
Phnom Penh bat virus (PPBV)	No	1969	Cambodia, Malaysia	*Cynopterus brachyotis* (Lesser short-nosed fruit bat), *Eonycteris spelaea* (dawn bat)	[24,30]
Rio Bravo virus (RBV) ^e^	Yes (fever)	1954	United States, Mexico, Trinidad	*Eptesicus fuscus* (big brown bat), ^f^ *Molossus rufus* (black mastiff bat), *Tadarida brasiliensis mexicana* (Mexican free-tailed bat)	[24,31,32,33,34,35]
Sokoluk virus (SOKV) ^g^	No	1970	Kyrgyzstan, Russia	*Pipistrellus* spp. bats, *Argasidae* spp. ticks	[36,37]
Tamana bat virus (TABV) ^h^	No	1973	Trinidad	*Pteronotus parnellii* (Parnell’s mustached bat)	[32]
Yokose virus (YOKV)	No	1971	Japan	*Miniopterus fuliginosus* (eastern bent-wing bat)	[38]

^a^ Restricted to species that have yielded isolates (serological data not considered); ^b^ Subtype of Phnom Penh bat virus; ^c^ Formerly known as Entebbe bat salivary gland virus; ^d^ Also known as *Chaerephon (Tadarida) limbata*; ^e^ Formerly known as bat salivary gland virus; ^f^ Also known as *Molossus ater*; ^g^ subtype of Entebbe bat virus; ^h^ Not classified as a NKV flavivirus by the International Committee on Taxonomy of Viruses (ICTV); it has been tentatively assigned to the genus *Flavivirus* and has no known arthropod vector.

**Table 2:** Geographic distribution, natural host range and clinical manifestations of rodent-associated NKV flaviviruses.

Virus	Human Disease	Year of First Isolation	Geographic Distribution	Natural Host Range ^a^	Reference
Apoi virus (APOIV)	Yes (encephalitis)	1954	Japan	*Apodemus* and/or *Clethrionomys* spp. ^b^	[24]
Cowbone Ridge virus (CRV)	No	1965	United States	*Sigmodon hispidus* (hispid cotton rat)	[39]
Jutiapa virus (JUTV)	No	1969	Guatemala	*Sigmodon hispidus* (hispid cotton rat)	[24]
Modoc virus (MODV)	Yes (meningitis)	1958	United States, Canada	*Peromyscus maniculatus* (deer mouse)	[35,40,41]
Sal Vieja virus (SVV)	No	1978	United States	*Peromyscus leucopus* (white-footed mouse)	[24]
San Perlita virus (SPV)	No	1971	United States	*Sigmodon hispidus* (hispid cotton rat)	[24]

^a^ Restricted to species that have yielded isolates (serological data not considered); ^b^ Isolated from pooled spleens harvested from five small Japanese field mice (*Apodemus argenteus*), one large Japanese field mouse (*Apodemus speciosus*) and one grey red-backed vole (*Myodes* (*Clethrionomys*) *rufocanus bedfordiae*).

**Table 3:** Summary of sequence data available for bat-associated NKV flaviviruses.

Virus	Sequence Data Available	Length of Genome (nt)	Length of 5′ UTR (nt)	Length of 3′ UTR (nt)	Length of Polyprotein (aa)	GenBank Accession No. ^a^
Batu Cave virus ^b^	ORF	-	*-*	-	3376	KJ469370
Bukalasa bat virus	Partial NS5	-	-	-	-	AF013365
Carey Island virus	Partial NS5	-	-	-	-	AF013368
Dakar bat virus	Partial NS3, Partial NS5	-	-	-	-	AF297462 (NS3), AF013371 (NS5)
Entebbe bat virus	ORF	-	119	-	3411	KP233893
Montana myotis leukoencephalitis virus	Genome	10,690	108	457	3374	NC_004119
Phnom Penh bat virus	ORF	-	*-*	-	3376	KJ469372
Rio Bravo virus	Genome	10,742	116	486	3379	JQ582840
Sokoluk virus ^c^	ORF	-	*-*	-	3413	NC_026624
Tamana bat virus ^d^	ORF	10,428	134	-	3350	AF346759
Yokose virus	Genome	10,857	150	429	3425	NC_005039

^a^ If multiple sequences have been deposited into the GenBank database, the accession number corresponding to the prototype isolate or longest sequence is shown (although two accession numbers are listed on occasion); ^b^ Subtype of Phnom Penh bat virus; ^c^ Subtype of Entebbe bat virus; ^d^ Not classified as a NKV flavivirus by the ICTV.

**Table 4:** Summary of sequence data available for rodent-associated NKV flaviviruses.

Virus	Sequence Data Available	Length of Genome (nt)	Length of 5′ UTR (nt)	Length of 3′ UTR (nt)	Length of Polyprotein (aa)	GenBank Accession No. ^a^
Apoi virus	ORF, 3′ UTR	-	-	576	3371	AF160193 (ORF), AF452050 (3′ UTR)
Cowbone Ridge virus	Partial NS3, Partial NS5	-	-	-	-	AF297461 (NS3), AF013370 (NS5)
Jutiapa virus	ORF	-	-	-	3374	KJ469371
Modoc virus	Genome	10,600	109	366	3374	NC_003635
Sal Vieja virus	Partial NS3, Partial NS5	-	-	-	-	AF297460 (NS3), AF013401 (NS5)
San Perlita virus	Partial NS5	-	-	-	-	AF013402

^a^ If multiple sequences have been deposited into the GenBank database, the accession number corresponding to the prototype isolate or longest sequence is shown (although two accession numbers are listed on occasion).

**Table 5:** Predicted cleavage sites in the polyproteins of NKV flaviviruses. **^a^**

Virus	Junction					
	VirionC/Anch (Dibasic)	anchC/prM (Signalase)	pr/M (Furin)	prM/E (Signalase)	E/NS1 (Signalase)	NS1/NS2A (Signalase Like)
APOIV	KGGRR↓GGKSV	PIALS↓AVVMN	TRTRR↓DVTIQ	APAYA↓STCVS	TGVVG↓EIGCM	GLVMA↓FDEEP
BCV	RKKQR↓SCGGS	GLGLG↓SVVRN	NRHRR↓SLDIA	TPAFG↓TQCVS	LGVVG↓DVGCA	GKVVA↓GDTHE
ENTV	ARKRR↓SSATH	GAACG↓IHVER	RRSRR↓SVEIT	APAYS↓THCTS	TGVGA↓ETGCA	SWVSA↓ADGRR
JUTV	AKKQR↓GGQVV	VLVLG↓MEVVR	IRERR↓SLPIA	APAIS↓TGCVG	TGVMG↓DHGCI	GLVMA↓CDGEV
MMLV	RKKQR↓SAKTV	ALMVA↓MEIEQ	ERAKR↓SLVIQ	APNLA↓TNCVS	TGVMG↓DQGCV	GLVSA↓QNEMS
MODV	KTKQR↓SAGWT	GTILS↓IEVVK	NRVRR↓AVNIA	LPSFA↓TNCVT	TGVMG↓DHGCV	GLVMA↓SDGEK
PPBV	RKKRR↓SRGES	GLGLG↓SVIRS	NRHRR↓SLDIA	TPAFG↓TQCVS	LGVVG↓DVGCA	GRVVA↓GDTHE
RBV	KKQRR↓GGTES	TGLMA↓MQVSQ	HRLKR↓SLSIT	APSYS↓TQCVN	TGVMG↓DHGCA	GLVYA↓GSMTA
SOKV	ARKRR↓SATLN	GTASA↓VHFNR	RRARR↓SVEIN	APAYS↓THCTN	VGVSA↓ETGCS	SWVSA↓GTGRK
^b^TABV	QKRQK↓SSGGY	MVIFC↓GYQSG	HRTRR↓SVTET	YLADA↓GHCHD	EVVAA↓DKYVL	NVVKA↓SKMNK
YOKV	KRKRR↓SSVSC	VTVGA↓LQIGR	RRNRR↓SVALT	APAYS↓THCTN	TGVGA↓EQACA	SWVSA↓GEGRM
	NS2A/NS2B (dibasic)	NS2B/NS3 (dibasic)	NS3/NS4A (dibasic)	NS4A/2K (dibasic)	2K/NS4B (signalase)	NS4B/NS5 (dibasic)
APOIV	ASRKR↓SGQRS	RSIQK↓SNTSF	AKGKR↓SGMTI	EGMQR↓TQVDS	AAVVA↓NEMGF	SENRR↓GVSSS
BCV	VFERR↓GVDVT	DQRQR↓SLLIM	ASMRK↓TSGLL	EGMQR↓TQIDS	IAVVA↓NEMRL	KSERR↓GLITS
ENTV	RTAKR↓SMDWT	YTSRR↓SNIMW	ATATR↓SMTTI	AGMQR↓STQDN	GLVAA↓NENGY	RGNRR↓GGGGT
JUTV	WPWRR↓SIRTT	PREQR↓SLIVY	GEMRR↓SVVME	EGMQR↓TQIDT	GMVVA↓NEMRW	KSQRR↓GIVTS
MMLV	QPSKR↓ATDYM	DGKRR↓SLYLL	AEKRR↓SSVLT	QGMQR↓TQIDT	LLVFA↓NEMRW	SPGRR↓GLSLS
MODV	PRHIR↓GVDYV	GKEQR↓SLIVY	AEMRR↓SSVWL	EGQQR↓TQIDT	GLVIA↓NELRW	TSNRR↓GICSS
PPBV	TFERR↓GVDVT	DQRQR↓SLLIM	ASMRK↓TSGLL	EGMQR↓TQIDS	IAVVA↓NEMRL	KSERR↓GLTTN
RBV	HRGQR↓ATDYT	DATQR↓SIIVF	AQMRR↓SGVLL	EGMQR↓TQIDS	VTVVA↓NEMRL	RSDRR↓GIVTS
SOKV	RVSRR↓SLDWT	YTSRR↓SNIIW	ASTTR↓SMINI	AGMQR↓SSQDN	GLIAA↓NENGY	QGNRR↓SGGGE
TABV	not identified	NLRDK↓SKGLI	DVNTR↓TRQNV	TQREK↓STGEV	YYILA↓DGEIL	TQRFR↓SSIFT
YOKV	NGKVR↓SIDWT	YTKQR↓SNILW	ATTTR↓SITAV	TGMQR↓SIQDN	ALIVA↓NENGY	QANRR↓GGTGS

^a^ GenBank Accession numbers for the sequences used in this analysis are listed in Table 3 and Table 4; ^b^ Prediction of TABV cleavage sites is difficult due to high divergence from other flaviviruses. See also de Lamballerie et al. [44], in which slightly different positions where predicted for some sites.

## Supplementary Materials

The following are available online at www.mdpi.com/1999-4915/9/5/154/s1, Figure S1: Phylogenetic tree for genus *Flavivirus* based on the E protein, Figure S2: Phylogenetic tree for genus *Flavivirus* based on the NS3 protein, Figure S3: Phylogenetic tree for genus *Flavivirus* based on the NS5 protein.
