# Supplementary material for: A Review of Flaviviruses that Have No Known Arthropod Vector
Source: Viruses. 2017 Jun 21;9(6):154. doi: 10.3390/v9060154 (PMC5490829; doi:10.3390/v9060154)

Supplementary Figure 1. Phylogenetic tree for genus *Flavivirus* based on the E protein. Complete polyprotein amino acid sequences were aligned using MUSCLE (24) and the E protein region extracted. A maximum likelihood phylogenetic tree was estimated using the Bayesian Markov chain Monte Carlo method implemented in MrBayes version 3.2.3 (23) sampling across the default set of fixed amino acid rate matrices, with ten million generations, discarding the first 25% as burn-in. The figure was produced using FigTree (http://tree.bio.ed.ac.uk/software/figtree/). The tree is midpoint-rooted and nodes are labelled with posterior probability values.

Supplementary Figure 2. Phylogenetic tree for genus *Flavivirus* based on the NS3 protein. See Supplementary Figure 1 caption for details.

Supplementary Figure 3. Phylogenetic tree for genus *Flavivirus* based on the NS5 protein. See Supplementary Figure 1 caption for details.

Supplemental Figure 1

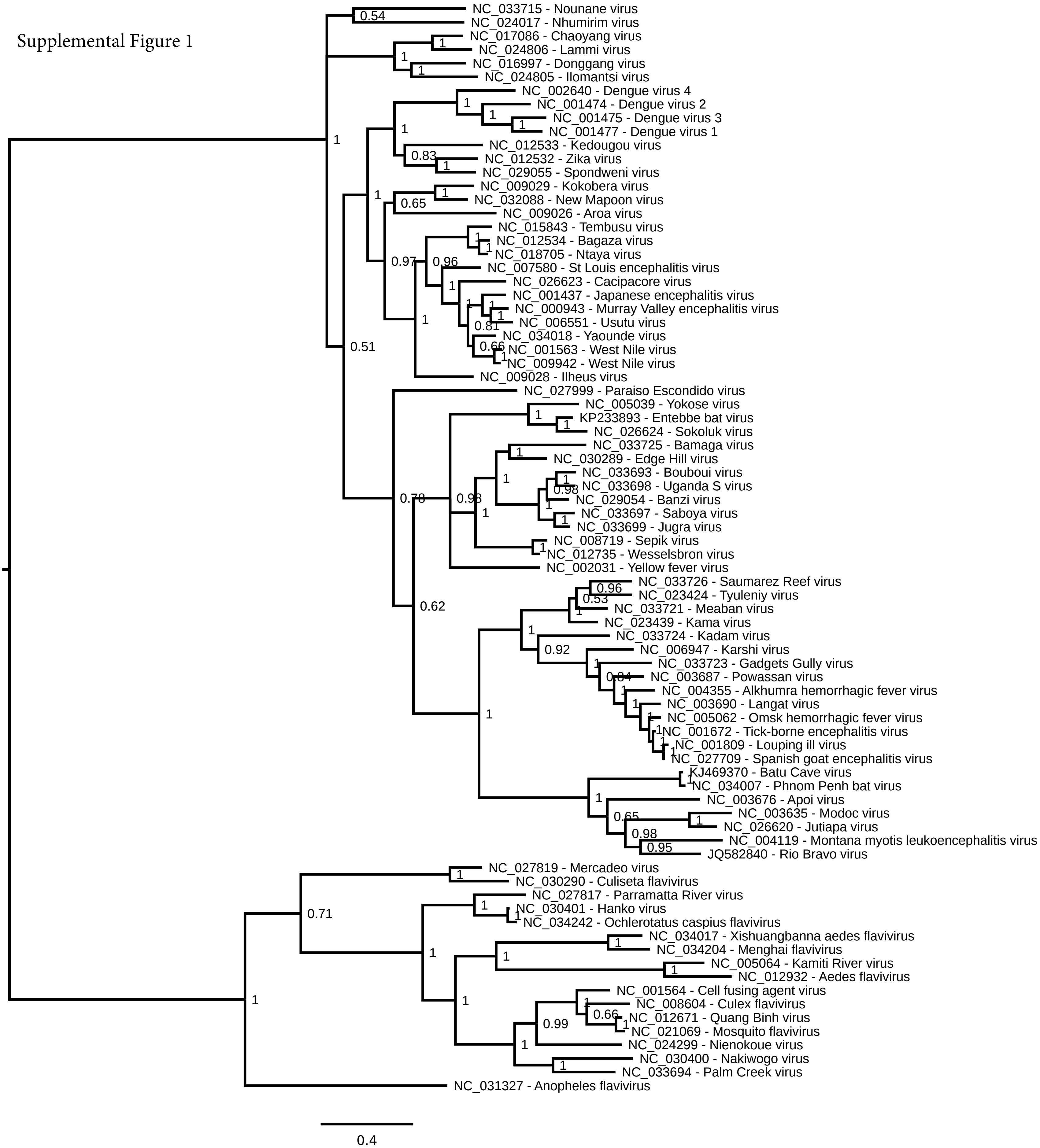

Supplemental Figure 2

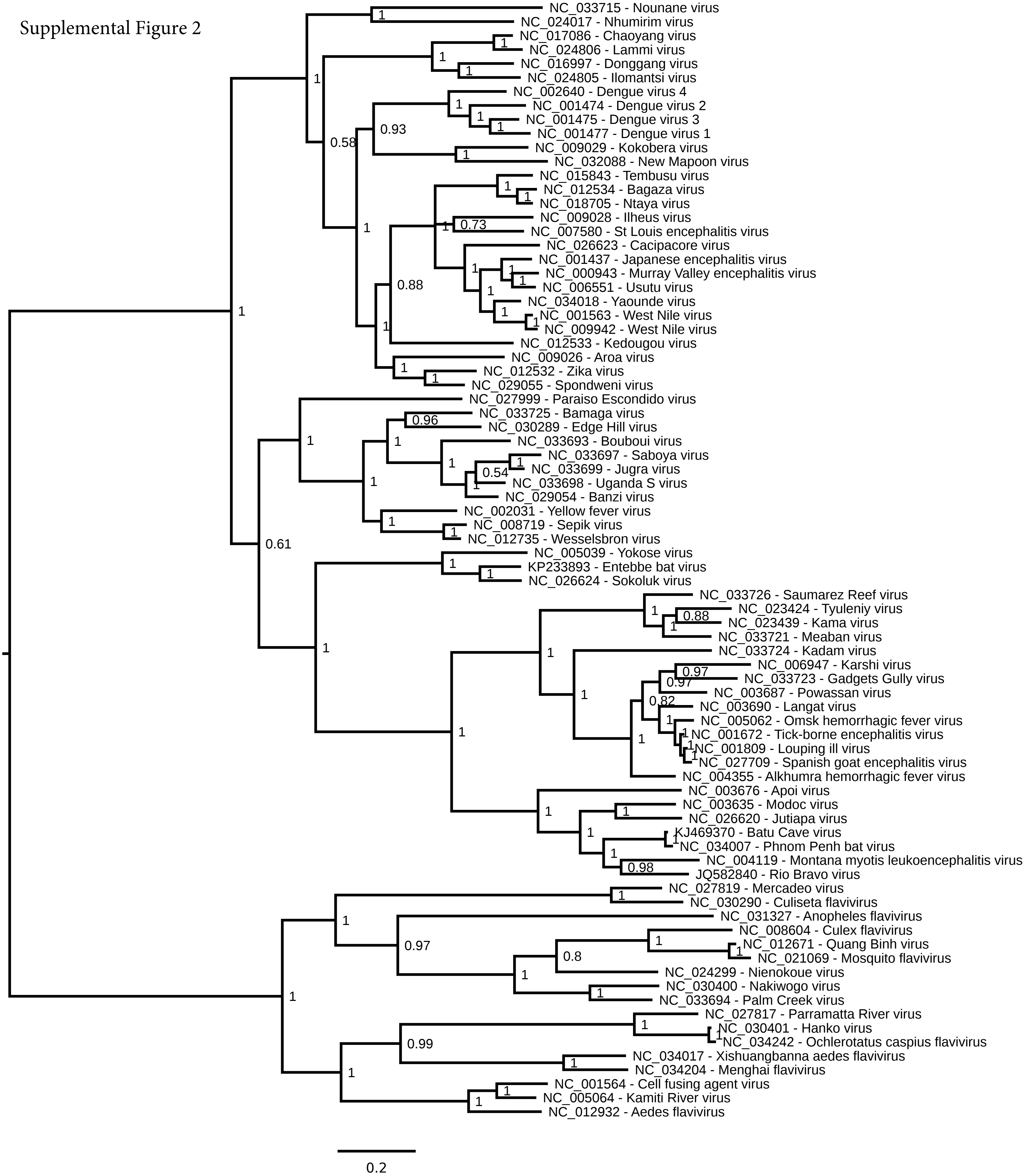

Supplemental Figure 3

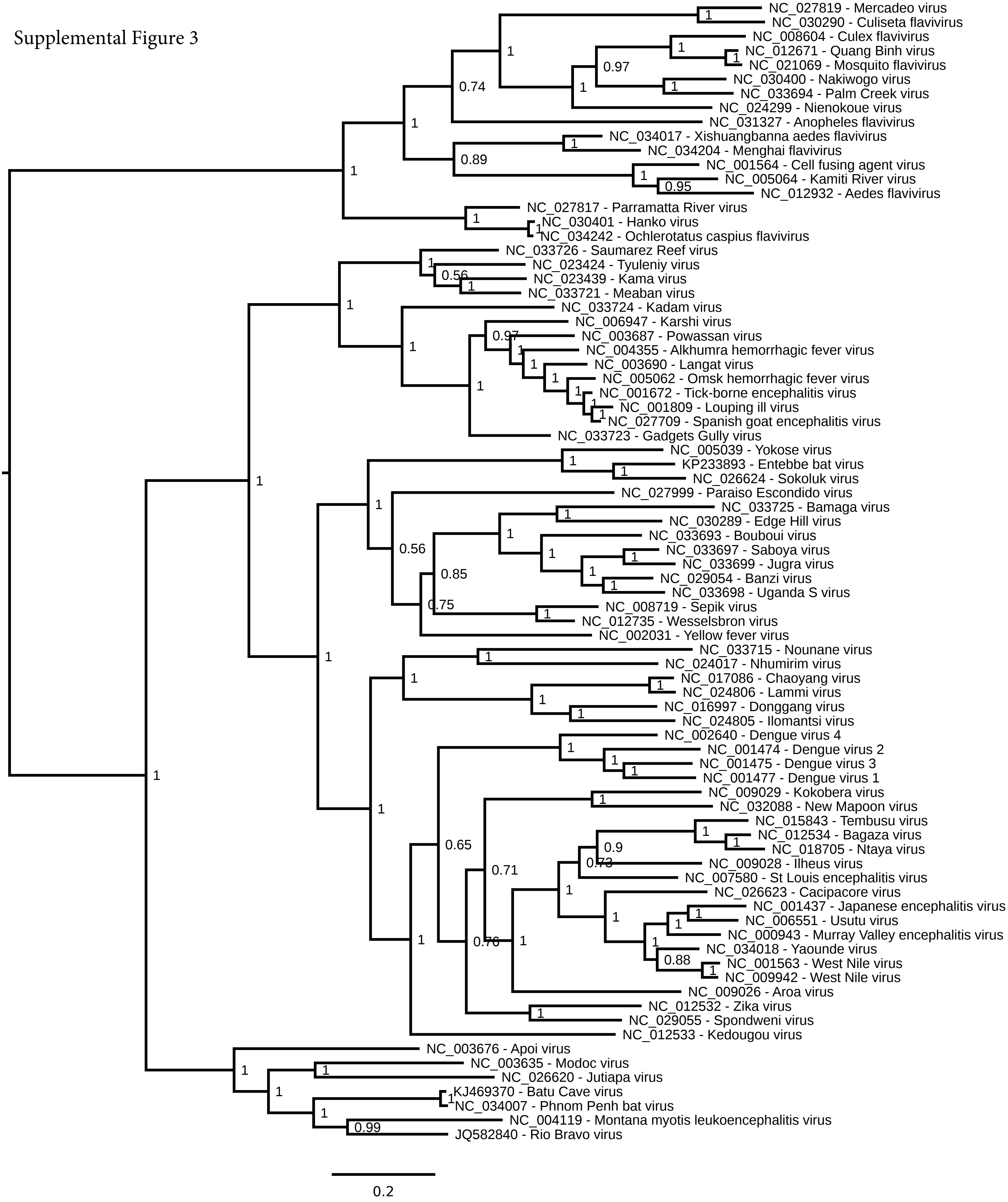

Supplement: Supplementary file 1 [file viruses-09-00154-s001.pdf]
